# Implementation of a Web-Based Program for Advance Care Planning and Evaluation of its Complexity With the Nonadoption, Abandonment, Scale-Up, Spread, And Sustainability (NASSS) Framework: Qualitative Evaluation Study

**DOI:** 10.2196/49507

**Published:** 2025-03-04

**Authors:** Doris van der Smissen, Maud A Schreijer, Lisette J E W C van Gemert-Pijnen, Rudolf M Verdaasdonk, Agnes van der Heide, Ida J Korfage, Judith A C Rietjens

**Affiliations:** 1 Department of Public Health Erasmus MC, University Medical Center Rotterdam Rotterdam The Netherlands; 2 Psychology, Health & Technology Faculty of Behavioural, Management and Social Sciences University of Twente Enschede The Netherlands; 3 Health Technology Implementation Faculty of Science and Technology University of Twente Enschede The Netherlands; 4 Department of Design, Organisation and Strategy Faculty of Industrial Design Engineering Delft University of Technology Delft The Netherlands

**Keywords:** eHealth, web-based intervention, implementation, sustainability, advance care planning, NASSS framework, nonadoption, abandonment, scale-up, spread, and sustainability framework, health communication, patient education, patient-centered care

## Abstract

**Background:**

The implementation of eHealth applications often fails. The NASSS (nonadoption, abandonment, scale-up, spread, and sustainability) framework aims to identify complexities in eHealth applications; the more complex, the more risk of implementation failure.

**Objective:**

This study aimed to analyze the implementation of the web-based advance care planning (ACP) program “Explore Your Preferences for Treatment and Care” using the NASSS framework.

**Methods:**

The NASSS framework enables a systematic approach to improve the implementation of eHealth tools. It is aimed at generating a rich and situated analysis of complexities in multiple domains, based on thematic analysis of existing and newly collected data. It also aims at supporting individuals and organizations to handle these complexities. We used 6 of 7 domains of the NASSS framework (ie, condition, technology, value proposition, adopters, external context, and embedding and adaptation over time) leaving out “organization,” and analyzed the multimodal dataset of a web-based ACP program, its development and evaluation, including peer-reviewed publications, notes of stakeholder group meetings, and interviews with stakeholders.

**Results:**

This study showed that the web-based ACP program uses straightforward technology, is embedded in a well-established web-based health platform, and in general appears to generate a positive value for stakeholders. A complexity is the rather broad target population of the program. A potential complexity considers the limited insight into the extent to which health care professionals adopt the program. Awareness of the relevance of the web-based ACP program may still be improved among target populations of ACP and among health care professionals. Furthermore, the program may especially appeal to those who value individual autonomy, self-management, and an explicit and direct communicative approach.

**Conclusions:**

Relatively few complexities were identified considering the implementation of the web-based ACP program “Explore Your Preferences for Treatment and Care.” The program is evidence-based, freestanding, and well-maintained, with straightforward, well-understood technology. The program is expected to generate a positive value for different stakeholders. Complexities include the broad target population of the program and sociocultural factors. People with limited digital literacy may need support to use the program. Its uptake might be improved by increasing awareness of ACP and the program among a wider population of potential users and among health care professionals. Addressing these issues may guide future use and sustainability of the program.

## Introduction

eHealth tools can be useful for health promotion, enhancing self-management skills for people with long-term conditions, and patient-physician communication [1]. The web-based program “Explore Your Preferences for Treatment and Care” [2] is aimed at supporting users in engaging in advance care planning (ACP). ACP is a communication process that enables persons to think about their goals and preferences for future treatment and care, to discuss these with their relatives or health care professionals, and to record these if appropriate [3]. The web-based ACP program guides users through 3 steps of ACP—exploration, discussion, and recording of preferences for future treatment and care (Textbox 1). A before and after study showed that the web-based ACP program increased ACP engagement among persons with a chronic disease and was perceived as usable, attractive, and comprehensible [4]. The program was embedded as a decision aid in the Dutch platform “Thuisarts” [5] in April 2020 (English version “GPinfo website” is in development). This platform is owned and hosted by the Dutch College of General Practitioners and provides evidence-based health-related information to the public.

**Main characteristics of the web-based advance care planning program “Explore Your Preferences for Treatment and Care” [2,4].**

**Content**

- Information about advance care planning (ACP).
- Thinking about values and quality of life.
- Communication about preferences with relatives and health care professionals.
- Appointing a health care representative.
- Recording of preferences in an advance directive.
- Reviewing the advance directive.
- References to information about specific diseases, patient organizations, and peer support opportunities.
- The content was based on a scoping review that explored the content, feasibility, and effectiveness of web-based ACP programs [6]; an interview study that identified information needs for web-based ACP of patients with chronic diseases and their relatives [7]; and meetings with a stakeholder group containing relevant stakeholders for the web-based ACP program including patients, relatives, and patient organizations [4].

**Structure**

- Interactive program; users can watch videos and click on additional information, users are asked questions regarding ACP and can save a document containing their responses.
- Stepwise approach to guide users through the ACP process.
- Embedment in information platform (Thuisarts website [English version: GPinfo website]) which is hosted and owned by the Dutch College of General Practitioners, and provides evidence-based health information.
- Accessible for free.
- Hyperlinks to external websites.
- Text-to-speech option.

**Development**

- Developed by researchers (DvdS, IJK, JACR, and AvdH) with expertise in shared decision-making, care at the end of life, and eHealth, in collaboration with stakeholder group including 1 patient, 2 relatives, representatives of the Dutch College of General Practitioners (Nederlands Huisartsen Genootschap), the Dutch Association for Kidney Patients (Nierpatiënten Vereniging Nederland), the Dutch Patient Association (Nederlandse Patiëntenvereniging), Agora (organization to promote the palliative approach), 1 expert in health communication of the Nivel (Netherlands institute for health services research), 1 expert in eHealth of the University of Twente (LvG-P), and 1 representative of Vital Innovators, an organization that conducts Social Return of Investment analyses.
- The development was funded by the Netherlands Health Organisation for Health Research and Development.

**Figure 1:**
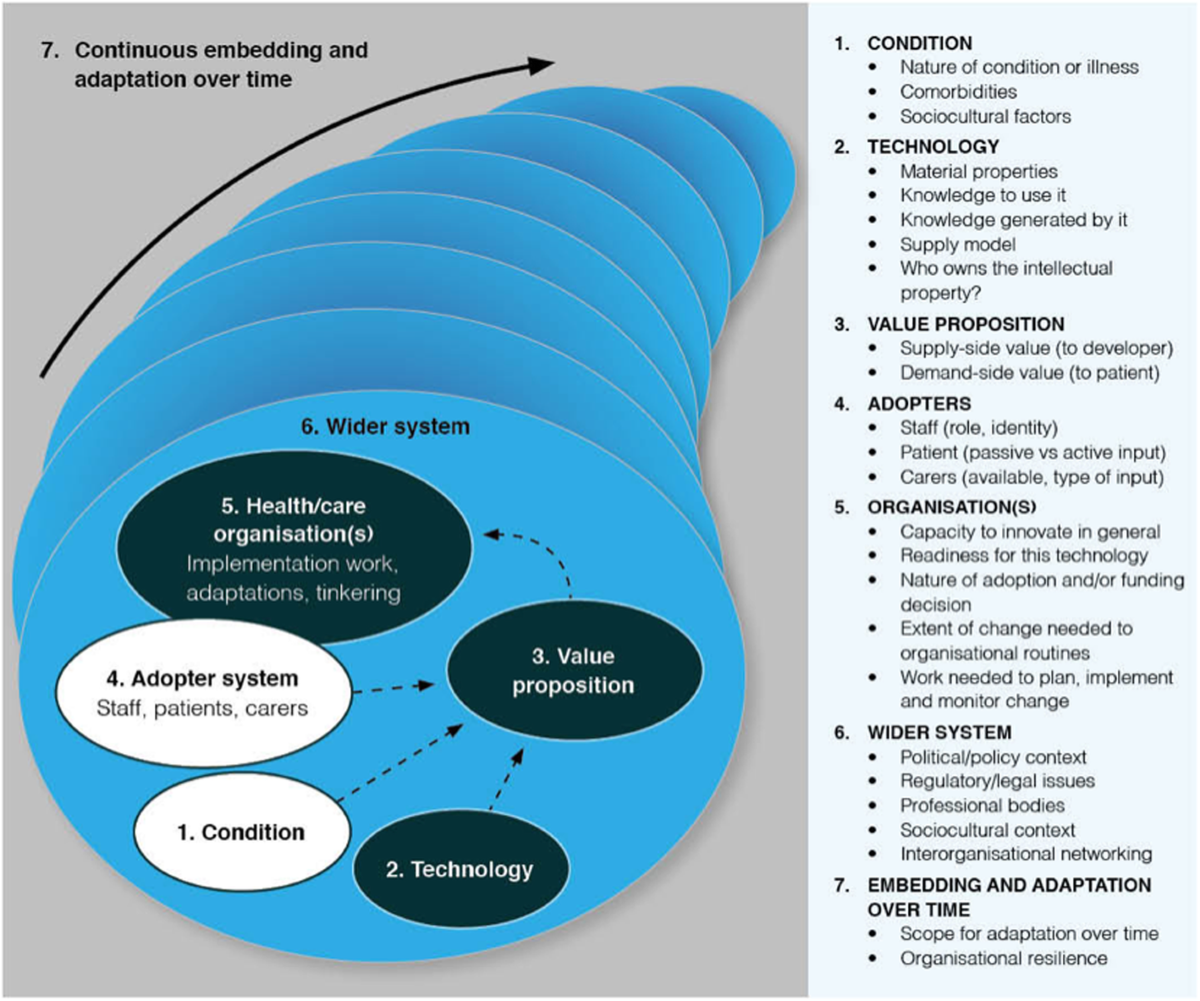
The nonadoption, abandonment, scale-up, spread, and sustainability (NASSS) framework [8] published under Creative Commons Attribution 4.0 International License [12].

Although many eHealth tools are developed, when they are implemented (ie, made available to end users), their sustainable implementation often fails [8]. To identify issues that may hamper the implementation of eHealth tools early, the NASSS (nonadoption, abandonment, scale-up, spread, and sustainability) framework has been developed by Prof T Greenhalgh and her team at Oxford University. The framework is aimed to encourage timely reflecting eHealth applications in health care and to systematically explore the chances of successful implementation of these eHealth applications by identifying complexities and reducing them [8]. NASSS stands for “nonadoption, abandonment, scale-up, spread, and sustainability over time,” which addresses the 5 possible reasons for eHealth implementation to fail [8]. The NASSS framework enables a systematic approach to explore complexities of eHealth tools regarding their implementation; the more complex, the more risk of uptake failure [8]. NASSS focuses at 7 key domains; condition, technology, value proposition, adopters, organization, external context, and embedding and adaptation over time (Figure 1) [8]. A domain can be classified as “simple” when it is straightforward, predictable, and only contains a few components (as in making a sandwich), “complicated” when it contains multiple interacting components (as in building a rocket), and “complex” when it is dynamic, unpredictable, and not easily disaggregated into constituent components (as in raising a child) [8,9]. The NASSS framework postulates that technologies with most domains classified as “simple” are more likely to be successfully implemented than technologies where most domains are classified as “complex” or “complicated.” Complexities in different domains can be interdependent, for example, when organizations have difficulty to adopt an eHealth technology, this may complicate adoption of the eHealth tool by staff as well [10,11]. We used the NASSS framework to identify complexities that may hamper sustainable implementation of the web-based ACP program.

## Methods

### Data Analysis

We evaluated the complexity of the web-based ACP program “Explore Your Preferences for Treatment and Care” [2] using the NASSS framework, following the approach of Abimbola et al [11] who also conducted an ex post (retrospective) thematic analysis.

First, we developed a multimodal dataset consisting of different existing, relevant data sources (Table 1). The dataset includes peer-reviewed and published studies describing the development and evaluation of the program [4,6,7], notes of stakeholder group meetings, and a social return on investment analysis in which the social costs (input) and benefits for stakeholders (outcomes) of the program were mapped.

Next, 2 researchers (DvdS an MAS) developed a data extraction form based on the nonadoption, abandonment, scale-up, spread, and sustainability complexity assessment tool (NASSS-CAT; IRIHS group); the NASSS CAT-LONG interview guide [13]. They used this form to extract relevant pieces of text from the data sources per NASSS domain, that is, (1) condition, (2) technology, (3) value proposition, (4) adopters, (5; previously 6) external context, (6; previously 7) embedding and adaptation over time. We considered the original domain 5, “organization,” not applicable because the web-based ACP program is not implemented in a specific health care organization and the hosting organization, the Dutch College of General Practitioners, did not have to change their routines to embed the program. Next, DvdS and MAS together with the wider research group conducted an initial thematic analysis of the extracted information to identify whether sufficient information per domain was available. They conducted 3 additional semistructured interviews to complete the dataset (1) with a representative of the Dutch college of general practitioners (the program host), (2) a general practitioner, and (3) a representative of the organization that funded the development of the web-based ACP program. The interview questions were based on the NASSS CAT-LONG interview guide [13]. Interviewees provided written informed consent for participating in an online interview in which questions were asked about attitudes toward the web-based ACP program related to the domains of the NASSS framework. Interviews were audio-recorded and transcribed verbatim.

Subsequently, DvdS and MAS together with the wider research group conducted a thematic analysis of the final dataset, using the NASSS framework as a lens, leading to a thematic description of complexities per domain. We used relevant ACP literature to further underpin the findings. The conclusion whether a domain is simple, complicated, or complex was based on discussion and eventually consensus among the members of the research group, where we weighed the potential impact and relevance of the different complexities.

Complexities in each of the 6 domains were classified as “simple,” “complicated,” or “complex.” Per domain, all relevant aspects were taken into consideration. An overall classification as “simple” does not require a total absence of room for improvement for a domain; however, most of its elements should be considered as simple. Based on the multimodal dataset and the classification in Multimedia Appendix 1 [8,9], DvdS and MAS prepared a classification per domain, which was discussed during meetings with all researchers (MAS, DvdS, JACR, IJK, and AvdH), until consensus was reached.

**Table 1:** Overview of the multimodal dataset used to analyze complexities of the web-based advance care planning program “Explore Your Preferences for Treatment and Care.”

Sources of information			NASSS^a^ framework domains										
			Condition		Technology		Value proposition		Adopters		External context		Embedding and adaptation over time
**Results and data from studies:**													
	Scoping review [6]: Aim: to determine the feasibility and effectiveness of 11 web-based ACP^b^ programs in 27 studies.					✓^c^				✓			
	Interview study [7]: Aim: identifying information needs for web-based ACP. Participants: 9 patients with a chronic disease and 7 relatives.					✓		✓					
	Pilot study [4]: Aim: assessing the usability and feasibility of the web-based ACP program. Participants: 3 health care professionals (2 general practitioners and 1 vascular surgeon) and 6 patients with a chronic disease.			✓		✓		✓					
	Evaluation study [4]: Aim: evaluation of ACP engagement (before using the web-based program vs 2 months after completion), usability, and users’ satisfaction (including comprehensibility). Participants: 147 patients with a chronic disease.			✓		✓		✓					
**Other available resources:**													
	Notes of stakeholder group meetings: Including researchers, patient organizations, patients, and relatives. Decisions were made concerning the program (eg, definition of target group and content of the program).	✓						✓		✓		✓	
	SROI^d^ analysis: The social costs and benefits for stakeholders, and mapped investments (input) and revenues (outcomes) of the program. The SROI methodology followed 9 steps that led to the complete cost-benefit model and the final calculated SROI ratio. Interviews were conducted with health care organizations, stakeholder group, patients and relatives, and a health insurance company.					✓		✓				✓	
	Content of the web-based program: Content, structure, layout, or assembly. Page with additional information (information about target group and purpose of ACP).	✓		✓									
	Platform of the web-based program (“Thuisarts”): Requirements of the program, consultations with the Thuisarts website. Guidelines of the Thuisarts website (eg, not saving personal data and maintenance web-based program).	✓		✓				✓		✓		✓	
	Publicity web-based program: Interviews with researchers and involved patients and relatives in magazines and the news. News articles.							✓		✓		✓	
	Number of visits to web-based ACP program							✓		✓			
	Financial information: Funder of the development of the web-based ACP program. Thuisarts website.					✓							
	Relevant literature	✓		✓		✓		✓		✓		✓	
**Newly collected data**													
	Interviews with 3 additional key stakeholders	✓		✓		✓		✓		✓		✓	

^a^NASSS: nonadoption, abandonment, scale-up, spread, and sustainability.

^b^ACP: advance care planning.

^c^✓: included in the dataset.

^d^SROI: social return on investment.

### Ethical Considerations

This study was approved by the Medical Research Ethics Committee of the Erasmus MC, University Medical Center Rotterdam on October 21, 2019 (MEC-2019-0590), confirming that the rules laid down in the Medical Research Involving Human Subjects Act do not apply to the interviews as conducted in this study. The study conforms with the International Committee of Medical Journal Editors’ recommendations for the conduct, reporting, editing, and publication and for the protection of research participants. The interview participants were provided with information about the aim and content of the interview. They provided written informed consent. Interview participants did not receive compensation. The authors confirm that all patient or personal identifiers have been removed or disguised so the persons described are not identifiable and cannot be identified through the details of the story.

## Results

### The Condition or Illness

The domain “condition or illness” considers to what extent the condition or illness is well-characterized, well-understood, and predictable and to what extent the condition or illness is influenced by comorbidities or sociocultural factors.

#### Target Group

The target group of the web-based ACP program is described as follows: “people can engage in ACP when they are healthy, but also when they become older or (chronically) ill, after having an accident or when nearing the end of life” [2]. The target group is hence not strictly demarcated, which is in line with recent recommendations [3,14,15]. During the development of the program, demarcating the target population was a recurring issue with some stakeholders promoting a focus on people with advanced illness and others promoting a broader scope. The target group ambiguity is also visible at the Thuisarts website, where the program is embedded: internal links to the program are included in pages about topics such as end-of-life care and dementia.

#### Sociocultural Factors Affecting Engagement in ACP

The web-based ACP program is aimed at individuals who are interested in ACP, regardless of their religion or culture. The program contains images and videos of both men (n=5) and women (n=3), persons with various ethnic backgrounds, and a wide range of ages (middle age to older adults) [2]. One video in the program includes a reference to a religious belief.

Nevertheless, the program is currently only available in the Dutch language and was only evaluated among Dutch-speaking persons. Multilingual support is not offered, which limits the accessibility of the program to non–Dutch-speaking individuals. The program fosters autonomy and self-management, and uses an explicit and direct communicative style [2]. This approach may be less appealing to people who consider the role of the family to be central in medical decision-making, and to those who prefer a less direct communication style [16]. This may limit the appeal of the program for those who consider the role of the family to be central in medical decision-making, and to those who prefer a less direct communication style [16].

#### Classification and Conclusion

The “condition or illness” domain is classified as complex given the rather undemarcated target population of the web-based ACP program and because the program may not address the ACP preferences of all potentially eligible persons.

### The Technology

The domain “the technology” encompasses a description of the web-based ACP program and the Thuisarts website, as well as user experiences with the technology.

#### The Web-Based ACP Program Embedded in Thuisarts Website

The web-based ACP program guides users through the process of ACP in three steps, that are (1) thinking about preferences for future treatment and care, (2) discussing these preferences with relatives and health care professionals, and (3) recording these preferences. Users are asked questions, and the answers to these can be saved in a document and printed. Users of the program are recommended to use this document during a conversation with a relative or a consultation with a health care professional. Users do not need to complete the program at once; they can access it at any preferred moment. The program is freely accessible without the need to register and data of users are not saved. Content maintenance of the program occurs through critical review of the program every 3 years by a stakeholder group of patients, relatives, health care professionals, and patient organizations.

The web-based ACP program is embedded in the Thuisarts website, a web-based platform containing information about health and disease, based on evidence-based guidelines (English version “GPinfo website” is in development). The platform is owned and maintained by the Dutch College of General Practitioners [5]. Approximately two-thirds of the Dutch population are familiar with the platform [17], and it has 6.6 million monthly visitors [18]. The content is written on the B1 level (easily readable and concise), and is offered in standardized formats [19], reading text out loud is enabled, and information is summarized in videos with subtitles. Every year, experts test whether the platform has the recommended level for web-based information (level AA) [20] and identified problems are solved [21].

#### User Experiences With the Technology

A qualitative pilot study showed that the web-based ACP program was acceptable and feasible for 6 interviewed patients with a chronic disease [4]. In an evaluation study among 147 members of an online research portal (who are expected to have at least some digital skills), including people with levels of low, middle, and high health literacy, the program was perceived as user-friendly (mean score of 70, SD 13, scale 0-100), attractive (mean 3.8, SD 0.7, scale 1-5), and comprehensible (mean 4.2, SD 0.6, scale 1-5) [4]. To support people with limited health- or digital literacy in using the program, people can watch videos and use a text-to-speech option to read the text aloud [4]. Furthermore, a clear and simple structure is used and the number of topics covered is not too large [4]. The texts are written on the B1 level (easily readable and concise) [19].

#### Classification and Conclusion

The “technology” domain is classified as simple because the web-based ACP program is freestanding and well-maintained, with straightforward, well-understood technology. An evaluation study showed that the program is perceived as user-friendly and comprehensible. However, people with limited health literacy or digital literacy might require help to use the program.

### The Value Proposition

The “value proposition” domain concerns whether a new technology is worth developing and for whom it may generate value. It includes demand-side value (value for the users) as well as supply-side value (value for the developers).

#### Value for Stakeholders

In total, 3 interviewed health care professionals indicated they considered the program as valuable to patients [4] and an evaluation study showed that after use of the web-based ACP program, people contemplated about ACP more often and indicated to feel more ready for ACP [4]. They considered the program to be user-friendly and were satisfied with the program [4]. This is in line with findings from a scoping review, which concluded that participants of web-based ACP programs generally consider these programs as easy to use, not burdensome, and feasible [6]. This review also indicated that web-based ACP programs are effective to improve ACP knowledge, communication about ACP, and documentation of ACP among program users [6].

Regarding the value for health care professionals, the funder of the web-based ACP program project and a general practitioner indicated in the additional interviews to consider the program as helpful to start ACP for health care professionals:

Some basic things are in there [in the program] which could be discussed: (…) like what is important to someone? That can help as a guidance in your conversation.

general practitioner

#### Costs

The web-based ACP program was developed in the context of a publicly funded research program and has no commercial purpose. The Dutch College of General Practitioners invests in long-term maintenance of the Thuisarts website and they will maintain the web-based ACP program technically as well.

#### Cost and Benefit Analysis

The web-based ACP program is freely accessible. The Thuisarts website is not aimed at making a profit. It is financed by the Dutch College of General Practitioners and receives no money from companies seeking financial gain, such as pharmaceutical companies. To better understand the relationship between the total investment (costs) and the expected social effects (benefits) of the web-based ACP program, a so-called social return on investment analysis was conducted [22]. This analysis showed that each investment in terms of money, time, and effort, is expected to generate a social return of approximately 1.7. This means that the program is expected to have a positive value for patients, relatives, health care professionals, and the health care system in terms of money or societal value, such as increased quality of life.

#### Classification and Conclusion

The “value proposition” domain is classified as simple because the technology is expected to generate a positive value for stakeholders (patients, relatives, health care professionals, and the health care system) while the benefits of the program are expected to exceed the costs. The Dutch College of General Practitioners invests in long-term maintenance of the Thuisarts website and they will maintain the web-based ACP program technically as well.

### The Adopters

The domain “adopters” focuses on the adoption and continued use of the technology by the groups of people who are intended to actually use the program or to refer potential users to the program, and what they need to use the program or to refer to it. The potential adopters of the web-based ACP program include patients, relatives, health care professionals, and the public in general.

#### Patients and the Public

**Figure 2:**
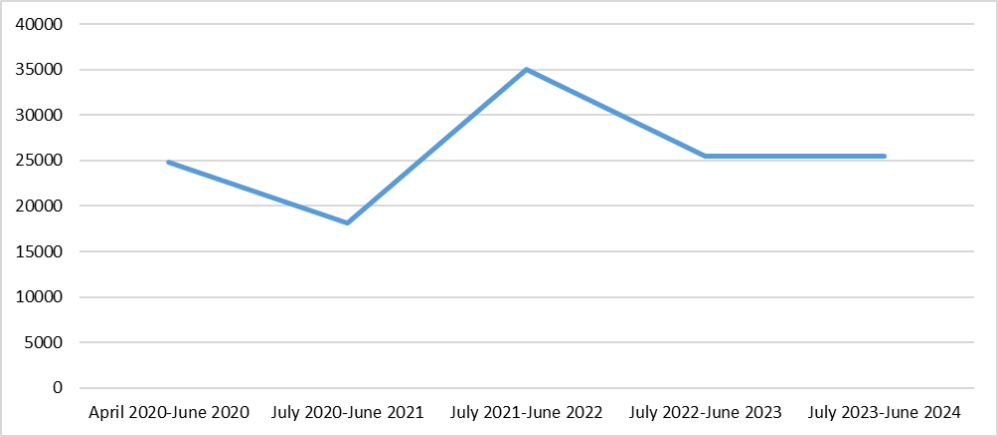
Visits to the web-based advance care planning program “Explore Your Preferences for Treatment and Care” from its launch (April 1, 2020) to June 30, 2024.

Patients with chronic disease and their relatives were asked about their information needs for ACP in a qualitative interview study [7]. They indicated the need for guidance in ACP, information about their disease and care, and information on how to communicate their preferences to relatives and health care professionals [7]. These needs were taken into account in the development of the web-based ACP program. In an evaluation study, users of the web-based ACP program indicated that the program helped them to consider ACP [4]. Users were generally satisfied with the amount of information in the program [4]. A few users mentioned that it was confronting to complete the program because of the difficulty of the topic and a large amount of information. In the first 3 months after its launch (April 1-June 30, 2020) the program was visited 24,849 times. From July 1, 2020, to June 30, 2021, it was visited 18,160 times; from July 1, 2021, to June 30, 2022, it was visited 35,026 times; from July 1, 2022, to June 30, 2023, it was visited 25,423 times; and from July 1, 2023, to June 30, 2024, it was visited 25,510 times. In total, it was visited 128,986 times (Figure 2).

More than 40 medical and patient organizations refer to the web-based ACP program on their websites. However, as described in the domain “condition or illness,” not everyone may be interested in engaging in ACP because of a preference to focus on their health and life in the present [23] or valuation of other principles than those central to the web-based ACP program, for example, valuing a central role of the family in medical decision making [16]. While the web-based ACP program is considered useful, it may require substantial commitment and effort of the user in terms of reflecting on their values and preferences for treatment and care.

#### Health Care Professionals

The web-based ACP program recommends participants to involve their health care professionals in the ACP process, for instance, to explain the medical situation, discuss patients’ preferences, or file an advance directive. It is known that most people access ACP information through health care professionals [24] and expect that the professional initiates ACP [25]. The Thuisarts website is a frequently used platform among health care professionals; 90% (1751 of 1946) of general practitioners are familiar with it and inform patients about it, and 73% (1421 of 1946) use it for information provision during their consultations [26]. Health care professionals were included in the stakeholder group and provided input during the development of the web-based ACP program. However, opinions about the web-based ACP program of health care professionals have not been extensively evaluated [4]. In total, 3 interviewed health care professionals considered the program to be of value [4]. However, several other studies have described that health care professionals may experience barriers in the initiation of ACP. ACP may be time-consuming, health care professionals report to lack the skills in initiating sensitive ACP conversations [25,27], and they may fear taking away patients’ hope [25,27]. Whether the web-based ACP program may support health care professionals to overcome these barriers remains to be studied.

#### Classification and Conclusion

The “adopters” domain is classified as simple. However, we identified a few complexities. While the web-based ACP program is potentially useful, it may require substantial commitment and effort of users. Furthermore, it remains to be studied whether the program can support health care professionals to overcome well-known barriers to ACP such as lack of time and fear of taking away patients’ hope.

### The External Context

The “external context” domain encompasses (1) the policy and political climate, (2) professional organizations and patient organizations, (3) the regulatory context, and (4) the economic context.

#### Policy and Political Climate

ACP is generally encouraged by organizations of health care professionals in the Netherlands, such as general practitioners, home care organizations, and hospitals. Many initiatives are taken to implement ACP in health care practice such as the development of ACP tools and manuals, websites for the public as well as health care professionals such as Palliaweb website, and ACP modules for electronic patient files [3,28-30]. In addition, the Dutch Ministry of Health, Welfare and Sport has spent €51 million (US $52.6 million) in the period 2014-2020 on research on palliative care including ACP [30] and initiated another research program on ACP in 2022 [31]. Furthermore, the Ministry has organized a public information campaign in 2022 to raise awareness for the importance of timely talking about death and dying [32,33].

#### Professional Organizations and Patient Organizations

Several patient organizations were involved in the development, evaluation, and implementation of the web-based ACP program. More than 40 organizations, including The Netherlands Patient Federation and the Dutch Association for Kidney Patients, as well as general practitioners and care organizations, referred to the web-based ACP program on their websites and in newsletters.

#### Regulatory Context

The regulatory context of ACP in general in the Netherlands is supportive regarding ACP. Several professional bodies have developed and provided guidelines for ACP [34], toolkits [35], and manuals for doctors [29,36]. Some of these specifically refer to the web-based ACP program [29,36]. Some materials are specifically developed for patients [37]. However, these ACP materials have different target groups, for instance, health care providers [29,34,36], patients in the palliative phase or at the end of life [3,14,15], or broader populations [3], and therefore it may not always be clear to whom they apply. Regarding the regulatory context of the web-based ACP program, users’ privacy is protected in compliance with data security and privacy requirements, by not requiring any log-in procedure and not recording any details of users.

#### Economic Context

The reimbursement of ACP for health care professionals is not clearly regulated.

#### Classification and Conclusion

The “external context” domain is classified as simple. Professional bodies are supportive toward the web-based ACP program and ACP in general. We found some complexities, such as different available manuals and tools for ACP containing different recommendations, and the unavailability of a treatment code for health care professionals. However, these external conditions are not likely to complicate the adoption of the web-based ACP program to a large extent.

### Embedding and Adaptation Over Time

#### Overview

The scope of the Thuisarts website is expanding. Information provision was focused on primary care in the past, but currently also includes information on secondary care. The Thuisarts website, including the web-based ACP program, is likely to be maintained in the future, with both technical and content updates. Content maintenance of the program occurs through a critical review of the program every 3 years by a stakeholder group of patients, relatives, health care professionals, and patient organizations. They will review compliance with (evolving) data security regulations, privacy requirements, and policies. If necessary, the program will be adapted. At this time, no changes in data protection regulations or policies are expected in the near future that would affect the program.

Our evaluation study showed that in general, participants had a positive user experience and were satisfied with the web-based ACP program [4]. To further improve this, we are systematically collecting informal feedback on the program and visitor numbers. These will be discussed during each 3-yearly review of the program by the stakeholder group. If necessary, they will make recommendations to adapt the program. To do so, the web-based ACP program will be adapted using an iterative design strategy of ongoing collecting informal feedback and the editorial team of the Thuisarts website adapting the program accordingly. Based on the feedback, user experience and satisfaction are aimed to be enhanced by improving the usability and feasibility of the web-based program.

#### Classification and Conclusion

We classify the “embedding and adaptation over time” domain as simple, since it is unlikely that the web-based ACP program as embedded in the Thuisarts website or its value are significantly going to change in the next years.

## Discussion

### Principal Results

To analyze the implementation of the web-based ACP program “Explore Your Preferences for Treatment and Care,” the NASSS framework was used to identify complexities within 6 domains. The analysis revealed no or little complexity in the domains “technology,” “value proposition,” “external context,” and “embedding and adaptation over time.” The program is evidence-based, freestanding, and well-maintained, with straightforward, well-understood technology. The program is expected to generate a positive value for different stakeholders as well as for the Thuisarts website. The analyses revealed some complexities in the “condition” domain, including the broad and rather undemarcated target population of ACP and the web-based ACP program and sociocultural factors that may limit users’ engagement. In the “adopters” domain, complexity entails that the program requires substantial commitment and effort by users with respect to reflecting on their preferences for future treatment and care. Some people with limited digital literacy may need support to use the program. Furthermore, it is yet unstudied to what extent health care professionals adopt the web-based ACP program. The program is embedded in the general practitioners’ platform “Thuisarts,” which is frequently used by general practitioners and the general public. The fact that the web-based ACP program is not embedded in a specific health care organization simplified its implementation, since no alignment regarding content or layout was required. On the other hand, embedding the program in the routine practice of a health care organization, could have boosted its use. Finally, we found some complexity in the “external context” domain. While the political and policy climate, regulatory context, professional organizations, and patient organizations are generally supportive regarding ACP, a national guideline for ACP and a treatment code to reimburse ACP could further increase willingness and ability of health care providers to engage in ACP. Overall, the analysis showed that the program has good potential for sustainable implementation, as it is expected to be continuously used in practice [8,38,39], will be updated if necessary, and will be maintained long-term.

### Comparison With Previous Work

The results of this study showed that the web-based ACP program “Explore Your Preferences for Treatment and Care” has relatively few complexities regarding the implementation. This may have several reasons. First, the user-centered design of the program included an extensive preparatory phase, including a scoping review to learn from existing web-based ACP programs [6], identification of the needs of patients with chronic disease and their relatives for web-based ACP in an interview study [7], and close collaboration with stakeholders including patients and relatives. Second, during the development of the program, we paid due attention to sustainable implementation in an existing, well-used platform—“Thuisarts.” The team of the Thuisarts website has expertise in developing information for the public with different levels of health literacy. It is remarkable that other web-based ACP programs often seem to have not paid due attention to implementation. Three recent reviews about web-based ACP programs [6,40,41] showed that studies have mainly focused on the development of web-based ACP programs and evaluation of their feasibility, usability, acceptability, and effectiveness, but most did not address their implementation. This is in line with reviews about implementing eHealth in general showing that research has mainly focused on the content and evaluation of eHealth tools, but less so took into account the external context and sustainable adoption in health care systems [8,10].

The results of the current study also indicated that health care professionals are an important group of adopters for the web-based ACP program. The web-based ACP program, following existing guidelines, recommends an active role of health care professionals in the ACP process of patients, for instance, to discuss diagnosis, prognosis, and relevant treatment and care options with the patient, and to document the ACP process in the medical file [3,4]. In the literature, several barriers are described that health care professionals may experience in the initiation of ACP, such as lack of time, lack of skills in conducting ACP conversations, and fear of taking away patients’ hope [25,27]. In the development of the web-based ACP program, the role of health care professionals was to a limited extent taken into account. First, the program is embedded in the general practitioners’ platform “Thuisarts,” which is frequently used by general practitioners [26] and the general public [17,18]. Furthermore, health care professionals were included in the stakeholder group and provided input during the development of the web-based ACP program. In the program, the user is encouraged to discuss and share their preferences with their health care professionals at several points [2]. However, the actual use of the web-based ACP program among health care professionals and their views and experiences regarding the program, are unstudied. Given the barriers that health care professionals may experience in the initiation of ACP [25,27], a patient-centered and community approach of ACP is important, in which patients can also take the initiative to engage in ACP themselves. This is central to the program, as the program supports the initiation of ACP, self-management of users, and encourages conversation with health care professionals about values, goals, and preferences.

The results indicated complexities considering the broad target population of ACP in the web-based ACP program. This complexity is a reflection of the evolving concept of ACP in the literature. Initially, ACP was conceptualized to be used for patients in the palliative phase and at the end of life [3,14,15]. More recently, an international consensus study on ACP recommended that individuals can engage in ACP in any stage of life, but that ACP can be more targeted when individuals’ health condition worsens or when their age increases [3]. This evolving concept of ACP aligns with the evolving concept of palliative care, whose definition has widened from a sole focus on the end of life to also including chronic illnesses [42] and a shift from in-hospital to community-based care [43]. Reaching the community with high-quality information and guidance about ACP through the Thuisarts website may therefore fit these developments.

### Strengths and Limitations

A strength of this study is our systematic approach of evaluating the web-based ACP program using the NASSS framework. It provided insight in its complexities that might hamper its implementation. Furthermore, we had a large dataset including publications about the development and evaluation of the web-based ACP program, notes of stakeholder meetings, and interviews with the program host, a general practitioner, and a funding agency representative. Insight into the complexities may be used to improve future sustainable implementation of the program, and may be used to improve other web-based programs as well.

A limitation is the start of the NASSS evaluation after the tool was developed and embedded in the Thuisarts website, which has limited the opportunity to adapt the program during its development. Some of the authors (DvdS, JACR, AvdH, and IJK) were the developers of the web-based ACP program, and therefore they could provide in-depth insight in the developmental process. However, although the authors aimed to analyze the implementation of the web-based ACP program objectively, there can be a bias in the evaluation.

### Recommendations to Improve Complexities of the Web-Based Advance Care Planning Program

To enhance sustainable implementation, we provide several recommendations to improve the identified complexities of the web-based ACP program.

First, to make the program available in other languages than Dutch. This may further enhance the accessibility of the program to people with different languages. For instance, an English version of the program could be placed on the GPinfo website, which is the English version of the Thuisarts website that is still in development. The web-based ACP program may be translated to other languages as well, to enhance its accessibility to people who are not proficient in Dutch. To improve cultural inclusiveness, future research should explore the needs of people from various cultural backgrounds with regard to ACP and if desired, the program could be culturally adapted or different versions of the program could be developed.

Second, to enhance accessibility of the web-based ACP program to persons with very limited health or digital literacy. The program was developed to assist individuals with limited health or digital literacy, by offering readable texts, text-to-speech options, and videos. However, individuals with very limited health or digital literacy may still experience barriers to use the program. To enhance accessibility of the program for these users, recommendations to complete the program together with a relative could be added to the program. Furthermore, a PDF version of the web-based ACP program could be developed, that could be printed by a relative or health care professional, to make the program also available “offline.”

Third, to inform health care professionals about the variance in readiness for ACP among patients. Not everyone may want to engage in ACP [44], for instance, due to different cultural backgrounds and different needs to consider treatment and care preferences. In addition, we recommend to inform and support health care professionals in how to conduct ACP with their patients. The web-based ACP program may help to overcome lack of time, since health care professionals can refer to the program, patients can prepare themselves for an ACP discussion on beforehand, and then the discussion with the health care professional can be scheduled.

Fourth, the concept of ACP as an ongoing process of considering preferences (also including healthy individuals) [3], could be embedded in national guidelines, and be routinely integrated in health care practice to reach a broader target group for ACP. National guidelines and availability of a treatment code may lower barriers in ACP for health care professionals, and may consequently lead to increased engagement with ACP for patients. Websites aimed at the general public could refer to the web-based ACP program, for instance, pages about shared decision-making and pages within the Thuisarts website about chronic diseases. Furthermore, we recommend to conduct public awareness campaigns about ACP (including web-based ACP programs), as these have been found to enhance awareness of ACP [45]. These may also help to enhance accessibility of ACP for people beyond the medical health care setting.

Finally, to ensure adaptation of the program to evolving data protection requirements. During critical review of the program, that will take place every 3 years by a stakeholder group of patients, relatives, health care professionals, and patient organizations, compliance with (evolving) data security regulations, privacy requirements, and policies should be carefully reviewed. If necessary, the program should be adapted. In addition, the strategy of the Thuisarts website, the host of the web-based ACP program, is aimed at adapting the information on the Thuisarts website to the evidence-based guidelines for general practitioners as developed by the Dutch College of General Practitioners. When guidelines change, the information at the Thuisarts website is adapted accordingly by their editorial team. Furthermore, the strategy of the Thuisarts website focuses on collaborating with health care professionals and policy makers, and on including patients’ perceptions in the information, to ensure the content is adapted to significant changes in health care landscapes or policies.

Since the domains of the NASSS framework may be interdependent, reducing complexity in a particular domain may consequently also reduce the complexity in other domains. For instance, in case of national guidelines on ACP, health care professionals may be more willing to initiate ACP with their patients and patients may be more ready to initiate ACP.

### Recommendations for Future Research

The following recommendations, based on the results and conclusions of the current study, can aid future implementation of comparable web-based ACP programs.

In order to improve the sustainable implementation of eHealth tools, in particular web-based ACP programs, first, we recommend to systematically and throughout the process (from idea to implementation) assess their complexities, with careful attention for all potential adopters, their external context, and their embedment and adaptation over time. The NASSS framework is suitable to conduct such evaluation, as well as other models that can be used for implementation or sustainability evaluation, such as the Normalization Process Theory (a framework to understand how interventions become embedded or “normalized” in health care settings) [46] and the Centre for eHealth Research Roadmap (CeHRes) [47,48]. These frameworks consider similar domains as the NASSS framework, sometimes ordered slightly differently, and can provide guidance for the sustainable development and implementation of eHealth technologies [46-48]. We expect that others who aim to implement similar web-based ACP programs and who apply comparable implementation frameworks, will benefit from the findings of this study and the recommendations. Yet, these should be contextualized for each application (eg, aligned with local policy frameworks and targeted to the target groups central in that program).

Second, we recommend identifying all relevant target groups of the eHealth tools, and explore their needs and preferences to ensure the tool meets their needs. For the web-based ACP program in particular, we recommend exploring health care professionals’ perspectives as well as their actual use and awareness of the program.

Third, we recommend assessing as well as improving the attractiveness of eHealth tools to potential users, including a culturally diverse population and to groups with low digital literacy.

Fourth, we recommend that research includes a long-term perspective regarding the implementation of eHealth tools. This may include evaluating the long-term effects as well as the identification of new scientific evidence that necessitates an update of the content of eHealth tools.

### Conclusions

Relatively few complexities were identified considering the implementation of the web-based ACP program “Explore Your Preferences for Treatment and Care.” The program is evidence-based, freestanding, and well-maintained, with straightforward, well-understood technology. The program is expected to generate a positive value for different stakeholders. Complexities include the broad target population of the program and sociocultural factors. People with limited digital literacy may need support to use the program. Its uptake might be improved by increasing awareness of ACP and the program among a wider population of potential users and among health care professionals. Addressing these issues may guide future use and sustainability of the program.

## Appendix

Multimedia Appendix 1 Domains and questions in the Non-adoption, abandonment, scale-up, spread, and sustainability (NASSS) framework (reproduced from Greenhalgh T et al [8], published under Creative Commons Attribution 4.0 International License [12].

## Abbreviations

ACP: advance care planning
CeHRes: Centre for eHealth Research Roadmap
NASSS: nonadoption, abandonment, scale-up, spread, and sustainability
NASSS-CAT: nonadoption, abandonment, scale-up, spread, and sustainability complexity assessment tool

## Data Availability

Research data will be available for verification for at least 15 years. All data will be retained and stored at the central network server of the Erasmus MC, University Medical Center Rotterdam, and can be accessed by those specifically granted access.
