# Supplementary material for: Implementation of a Web-Based Program for Advance Care Planning and Evaluation of its Complexity With the Nonadoption, Abandonment, Scale-Up, Spread, And Sustainability (NASSS) Framework: Qualitative Evaluation Study
Source: JMIR Aging. 2025 Mar 4;8:e49507. doi: 10.2196/49507 (PMC11920655; doi:10.2196/49507)
Supplement: Multimedia Appendix 1 [file aging_v8i1e49507_app1.pdf]

Multimedia Appendix 1

Domains and questions in the Non-adoption, abandonment, scale-up, spread, and sustainability (NASSS) framework *(reproduced with permission)* [8].

Domain/question	Simple	Complicated	Complex
**Domain 1: The condition or illness**			
1A. What is the nature of the condition or illness?	Well-characterized, well-understood, predictable	Not fully characterized, understood, or predictable	Poorly characterized, poorly understood, unpredictable, or high risk
1B. What are the relevant sociocultural factors and comorbidities?	Unlikely to affect care significantly	Must be factored into care plan and service model	Pose significant challenges to care planning and service provision
**Domain 2: The technology**			
2A. What are the key features of the technology?	Off-the-shelf or already installed, freestanding, dependable	Not yet developed or fully interoperable; not 100% dependable	Requires close embedding in complex technical systems; significant dependability issues
2B. What kind of knowledge does the technology bring into play?	Directly and transparently measures [changes in] the condition	Partially and indirectly measures [changes in] the condition	Link between data generated and [changes in] the condition is currently unpredictable or contested
2C. What knowledge and/or support is required to use the technology?	None or a simple set of instructions	Detailed instruction and training needed, perhaps with ongoing helpdesk support	Effective use of technology requires advanced training and/or support to adjust to new identity or organizational role
2D. What is the technology supply model?	Generic, “plug and play,” or COTS[a] solutions requiring minimal customization; easily substitutable if supplier withdraws	COTS solutions requiring significant customization or bespoke solutions; substitution difficult if supplier withdraws	Solutions requiring significant organizational reconfiguration or medium-to large scale-bespoke solutions; highly vulnerable to supplier withdrawal
**Domain 3: The value proposition**			
3A. What is the developer’s business case for the technology (supply-side value)?	Clear business case with strong chance of return on investment	Business case underdeveloped; potential risk to investors	Business case implausible; significant risk to investors
3B. What is its desirability, efficacy, safety, and cost effectiveness (demand-side value)?	Technology is desirable for patients, effective, safe, and cost effective	Technology’s desirability, efficacy, safety, or cost effectiveness is unknown or contested	Significant possibility that technology is undesirable, unsafe, ineffective, or unaffordable
**Domain 4: The adopter system**			
4A. What changes in staff roles, practices, and identities are implied?	None	Existing staff must learn new skills and/or new staff be appointed	Threat to professional identity, values, or scope of practice; risk of job loss

Domain/question	Simple	Complicated	Complex
4B. What is expected of the patient (and/or immediate caregiver)—and is this achievable by, and acceptable to, them?	Nothing	Routine tasks, eg, log on, enter data, converse	Complex tasks, eg, initiate changes in therapy, make judgments, organize
4C. What is assumed about the extended network of lay caregivers?	None	Assumes a caregiver will be available when needed	Assumes a network of caregivers with ability to coordinate their input
**Domain 5: The organization**			
5A. What is the organization's capacity to innovate?	Well-led organization with slack resources and good managerial relations; risk taking encouraged	Limited slack resources; suboptimal leadership and managerial relations; risk taking not encouraged	Severe resource pressures (eg, frozen posts); weak leadership and managerial relations; risk taking may be punished
5B. How ready is the organization for this technology-supported change?	High tension for change, good innovation-system fit, widespread support	Little tension for change; moderate innovation-system fit; some powerful opponents	No tension for change; poor innovation-system fit; many opponents, some with wrecking power
5C. How easy will the adoption and funding decision be?	Single organization with sufficient resources; anticipated cost savings; no new infrastructure or recurrent costs required	Multiple organizations with partnership relationship; cost-benefit balance favorable or neutral; new infrastructure (eg, staff roles, training, kit) can mostly be found from repurposing	Multiple organizations with no formal links and/or conflicting agendas; funding depends on cost savings across system; costs and benefits unclear; new infrastructure conflicts with existing; significant budget implications
5D. What changes will be needed in team interactions and routines?	No new team routines or care pathways needed	New team routines or care pathways that align readily with established ones	New team routines or care pathways that conflict with established ones
5E. What work is involved in implementation and who will do it?	Established shared vision; few simple tasks, uncontested and easily monitored	Some work needed to build shared vision, engage staff, enact new practices, and monitor impact	Significant work needed to build shared vision, engage staff, enact new practices, and monitor impact
**Domain 6: The wider context**			
6A. What is the political, economic, regulatory, professional (eg, medicolegal), and sociocultural context for program rollout?	Financial and regulatory requirements already in place nationally; professional bodies and civil society supportive	Financial and regulatory requirements being negotiated nationally; professional and lay stakeholders not yet committed	Financial and regulatory requirements raise tricky legal or other challenges; professional bodies and lay stakeholders unsupportive or opposed
**Domain 7: Embedding and adaptation over time**			
7A. How much scope is there for adapting and coevolving the technology and the service over time?	Strong scope for adapting and embedding the technology as local need or context changes	Potential for adapting and coevolving the technology and service is limited or uncertain	Significant barriers to further adaptation and/or coevolution of the technology or service
7B. How resilient is the organization to handling critical events and adapting to unforeseen eventualities?	Sense making, collective reflection, and adaptive action are ongoing and encouraged	Sense making, collective reflection, and adaptive action are difficult and viewed as low priority	Sense making, collective reflection, and adaptive action are discouraged in a rigid, inflexible implementation model

[a]COTS: customizable, off-the-shelf.
